# Detection of *Burkholderia* in the seeds of *Psychotria punctata* (Rubiaceae) – Microscopic evidence for vertical transmission in the leaf nodule symbiosis

**DOI:** 10.1371/journal.pone.0209091

**Published:** 2018-12-14

**Authors:** Arne Sinnesael, Sharon Eeckhout, Steven B. Janssens, Erik Smets, Bart Panis, Olivier Leroux, Brecht Verstraete

**Affiliations:** 1 Plant Conservation and Population Biology, Department of Biology, KU Leuven, Leuven, Belgium; 2 Botanic Garden Meise, Meise, Belgium; 3 Department of Biology, Ghent University, Ghent, Belgium; 4 Naturalis Biodiversity Center, University of Leiden, Leiden, the Netherlands; 5 Bioversity International, Leuven, Belgium; 6 Natural History Museum, University of Oslo, Oslo, Norway; Friedrich Schiller University, GERMANY

## Abstract

**Background and aims:**

The bacterial leaf nodule symbiosis is a close interaction between endophytes and their plant hosts, mainly within the coffee family. The interaction between Rubiaceae species and *Burkholderia* bacteria is unique due to its obligate nature, high specificity, and predominantly vertical transmission of the endophytes to the next generation of host plants. This vertical transmission is intriguing since it is the basis for the uniqueness of the symbiosis. However, unequivocal evidence of the location of the endophytes in the seeds is lacking. The aim of this paper is therefore to demonstrate the presence of the host specific endophyte in the seeds of *Psychotria punctata* and confirm its precise location. In addition, the suggested location of the endophyte in other parts of the host plant is investigated.

**Methods:**

To identify and locate the endophyte in *Psychotria punctata*, a two-level approach was adopted using both a molecular screening method and fluorescent *in situ* hybridisation microscopy.

**Key results:**

The endophytes, molecularly identified as *Candidatus* Burkholderia kirkii, were detected in the leaves, vegetative and flower buds, anthers, gynoecium, embryos, and young twigs. In addition, they were *in situ* localised in leaves, flowers and shoot apical meristems, and, for the first time, in between the cotyledons of the embryos.

**Conclusions:**

Both independent techniques detected the host specific endophyte in close proximity to the shoot apical meristem of the embryo, which confirms for the first time the exact location of the endophytes in the seeds. This study provides reliable proof that the endophytes are maintained throughout the growth and development of the host plant and are transmitted vertically to the offspring.

## Introduction

Plants interact with a broad range of endophytic microorganisms, ranging from fungi, such as arbuscular mycorrhizal fungi (AMF), to bacteria, including rhizobia [1–5]. Most of these interactions are neutral or mutualistic, with only a small percentage being pathogenic [2]. However, endosymbionts are not limited to the rhizosphere but are also detected in above-ground plant organs such as stems and leaves. The term ‘endophyte’ as defined by Partida-Martínez and Heil [3] is used in this study to refer to endosymbionts occurring in plant tissue without negatively affecting the host plant.

Bacterial leaf nodule symbiosis is an intimate endosymbiosis in which endophytes are housed in galls or ‘nodules’ in the leaves of several species in the flowering plant families Dioscoreaceae, Primulaceae, and Rubiaceae [6,7]. In the latter two families, the endosymbiosis is suggested to be unique due to the presence of vertical transmission, the obligate nature, and the high specificity of the interaction [6–9]. The presence of vertical transmission in combination with possible ecological advantages may transform beneficial interactions into long-term and possibly obligate mutualisms [2,10]. Bacterial leaf nodule symbiosis evolved into a highly specific and obligate endosymbiosis in which one specific host plant species interacts with one single bacterial species [7–9,11–12]. This leaf symbiosis is the most prevalent in Rubiaceae, where the endophytes were identified as *Burkholderia* species [6–9,13–18].

Papers like von Faber [19], Lersten and Horner [20,21], and Miller [6] are the most extensive microscopic reports on the nodulated species *Pavetta zimmermanniana* [19], *Psychotria bacteriophila* [20–21], and *Psychotria kirkii* [6] (the latter two being synonyms of *Psychotria punctata* [22]). These studies not only detected bacterial microorganisms in leaf nodules, but also found them in other plant structures such as vegetative and reproductive buds, sepals [6,19–21], and pyrenes (i.e., the stones of fleshy drupes containing the seeds) [19]. Although, vertical transmission of the endophytes to the next generation was suggested by von Faber [19]–based on the detection of bacterial microorganisms in the gynoecium (micropyle region) and the pyrenes (cavity surrounding the embryo)–to date, strong evidence is still lacking. Van Oevelen et al. [18] was the first to molecularly identify the endophyte in the leaf nodules of *Psychotria punctata* as *Candidatus* Burkholderia kirkii and Lemaire et al. [12] detected endophytic DNA in seeds, flowers, shoots, and leaves. Despite this progress, the exact location of *Candidatus* Burkholderia kirkii still remains unclear.

Here, we combine two methods (i.e., molecular screening and fluorescent *in situ* hybridisation) to study the location of the host specific endophyte, *Candidatus* Burkholderia kirkii, in the seeds of the nodulated host plant *Psychotria punctata*. We hypothesize that, if the bacterial endophytes are vertically transmitted, they should ideally be located in close proximity to the embryonic apical shoot meristem as this facilitates their transmission to the vegetative and reproductive structures of the next generation of host plants. In addition to the localisation of the endophytes in the seeds, we also screened other suggested locations of the bacterial endophytes within the host plant using the same approach.

## Materials and methods

### Plant material

Leaves, vegetative buds, flower buds, flowers, pyrenes, twigs, and roots of *Psychotria punctata* were obtained from plant specimens grown at the Botanic Garden Meise (accession numbers BR19536779, BR20010513-92, BR20001943-58, BR19951273-22, and BR20021526-47).

### Molecular analysis

For the molecular analysis, all plant structures, sampled in three-fold, were surface sterilised. Leaves, one-year-old and older lignifying twigs, roots, flowers, vegetative and flower buds were surface sterilised with 70% (v/v) ethanol and 1.6% (w/v) sodium hypochlorite for ten seconds each and the samples were subsequently rinsed with sterile water [7,12,14–17]. Pyrenes were treated with 70% (v/v) ethanol for three minutes and 1.6% (w/v) sodium hypochlorite with 0.1% (v/v) Tween20 for 20 minutes before rinsing them with sterile water. Anthers and gynoecia were dissected from the flowers, whereas endosperm, sclerified endocarp and embryos were prepared from the pyrenes. To remove the embryo, an incision was made along the ventral intrusion at the opposite side of the funicle. This incision facilitated the rupture of the pyrene and the isolation of the embryo from the embryonic cavity. For smaller plant structures, multiple samples of the same plant individual were combined, i.e., five gynoecia, ten anthers, and 15 embryos.

Plant tissues were ground in liquid nitrogen with a tissue homogeniser and their total genomic DNA was extracted using a modified cetyltrimethylammonium bromide (CTAB) protocol [23]. Polymerase chain reactions (PCR) were performed using 16S rDNA [15], *recA* and *gyrB* [24] bacterial primers, specifically designed for *Burkholderia* species. A positive control with *Burkholderia caledonica* and a negative control were included in each PCR run. The amplified products were visualised using gel electrophoresis and the positive results were purified and sequenced in both directions by Macrogen sequencing facilities (Macrogen Europe, Amsterdam, Netherlands). The raw sequences were assembled and screened for potential sequencing errors in Geneious v9.1.8 (Biomatters, Auckland, New Zealand). After assemblage, the consensus sequences were compared to sequences present on GenBank with the BLAST program (www.ncbi.nlm.nih.gov/BLAST) and were attributed to species level based on 99% or more sequence similarity [25].

### Microscopic analysis

The samples (i.e., three leaves, five embryos, three vegetative buds, and three flower buds) for microscopic analysis were fixed in 4% (v/v) formaldehyde in PEM buffer (100 mM 1,4-piperazinediethanesulfonic acid, 10 mM MgSO_4_, and 10 mM ethylene glycol tetra-acetic acid, pH 6.9) for two hours under vacuum. After washing in phosphate-buffered saline (PBS, Na_2_HPO_4_ 0.148 g, KH_2_PO_4_ 0.043 g, NaCl 0.72 g, NaN_3_ 0.9 g in 100 mL distilled water, pH 7.1), the samples were dehydrated using a graded ethanol series (30, 50, 70, 85, 100% (v/v)). Subsequently, they were gradually impregnated with and embedded in LR White acrylic resin (medium grade, London Resin Company, UK) using polypropylene embedding moulds and polymerized at 37°C for three days. Semi-thin sections of 350 nm were cut using a diamond knife mounted on a Leica UC7 ultramicrotome (Leica Microsystems, Vienna) and of each biological sample between five and ten sections were collected on polylysine-adhesion slides (Carl Roth, Germany).

As the FISH probe can only detect endophytes at the surface of sections, we also employed a ‘bacteria isolating’ technique [19] to collect endophytes from the outer surface of the embryo. To this end, three freshly excised embryos were submerged for five minutes in one droplet of sterile water on polylysine-adhesion slides. After air-drying the slides, the residue left by the embryos was fixed in 4% (v/v) formaldehyde in PEM buffer for 30 minutes and rinsed with PBS and sterile water. This ‘bacteria isolating’ technique was replicated three times.

Fluorescent *in situ* hybridisation (FISH) was performed as outlined by Daims et al. [26] using the 5’-Alexa-555-labelled Burkho primer (Thermo Fisher Scientific, US), a specific primer designed for *Burkholderia* [27]. The slides were mounted in Citifluor AF2 anti-fade agent (Agar Scientific, UK) and observations were made with a Nikon Eclipse Ni-U microscope equipped with a Nikon DS-Fi1c camera (Nikon, Japan) and the following filter cubes: FITC (excitation 480/20 BP; dichroic mirror 505 LP; emission 410 LP) referred to as the green channel and TRITC (excitation 535/30 BP; dichroic mirror 565 LP; emission 580 LP 541/572) referred to as the red channel. The green channel was used to observe plant tissue autofluorescence, while the red channel visualised the 5’-Alexa-555-labeled probe (excitation 555 nm, emission 580 nm). The use of merged epifluorescence images of both channels facilitated interpretation of the tissue-specific localisation of the endophyte. The hybridisation protocol of Daims et al. [26] with the Burkho probe was successfully tested on leaf cross sections through a bacterial leaf nodule.

After observation of FISH labelling, cover slips were carefully removed, and the slides were thoroughly rinsed with demineralised water to remove the anti-fade agent. Subsequently, the slides were stained with 1% (w/v) toluidine blue O (Merck, Germany) (TBO) in 1% (w/v) Na_2_B_4_O_7_ for 20 seconds at 50°C. After rinsing with demineralised water, slides were mounted with DePeX (VWR international, Belgium). Observations were made with a Nikon Eclipse Ni-U bright field microscope equipped with a Nikon DS-Fi1c camera.

## Results

### Molecular analysis

Endophytic DNA was detected in the leaves, vegetative buds, flower buds, anthers, gynoecia, embryos, and twigs with the specific *Burkholderia* primers 16S rDNA, *recA*, and *gyrB* (Table 1). *Burkholderia* DNA was not detected in the roots, the endocarp, nor in the endosperm of the seeds (Table 1). All the sequences were identified with BLAST (99% threshold) as *Candidatus* Burkholderia kirkii (S1 Table).

**Table 1:** Results of the PCRs with the specific primers for the genus *Burkholderia*. Of each plant structure, three biological replicates were tested. If the sequences were identified as *Candidatus* Burkholderia kirkii, the presence of the host specific endophyte is indicated with a +, otherwise the absence is indicated with a–in the last column.

Plant structure	16S	*recA*	*gyrb*	Identified as *Candidatus* Burkholderia kirkii
Leaves	3/3	3/3	3/3	+
Vegetative buds	3/3	3/3	3/3	+
Flower buds	3/3	3/3	3/3	+
Anthers	2/3	3/3	3/3	+
Gynoecia	3/3	3/3	3/3	+
Endocarp	0/3	0/3	0/3	–
Endosperm	0/3	0/3	0/3	–
Embryos	3/3	3/3	3/3	+
Twigs (second internode)	2/3	3/3	3/3	+
Twigs (lignifying, older sample)	3/3	3/3	3/3	+
Roots	0/3	0/3	0/3	–

Based on these results, plant structures for detailed microscopic analysis were determined, which included leaves, vegetative buds (*inter alia* shoot apical meristem), flower buds (*inter alia* gynoecium and anthers), and embryos.

### Fluorescent *in situ* hybridisation

**Fig 1:**
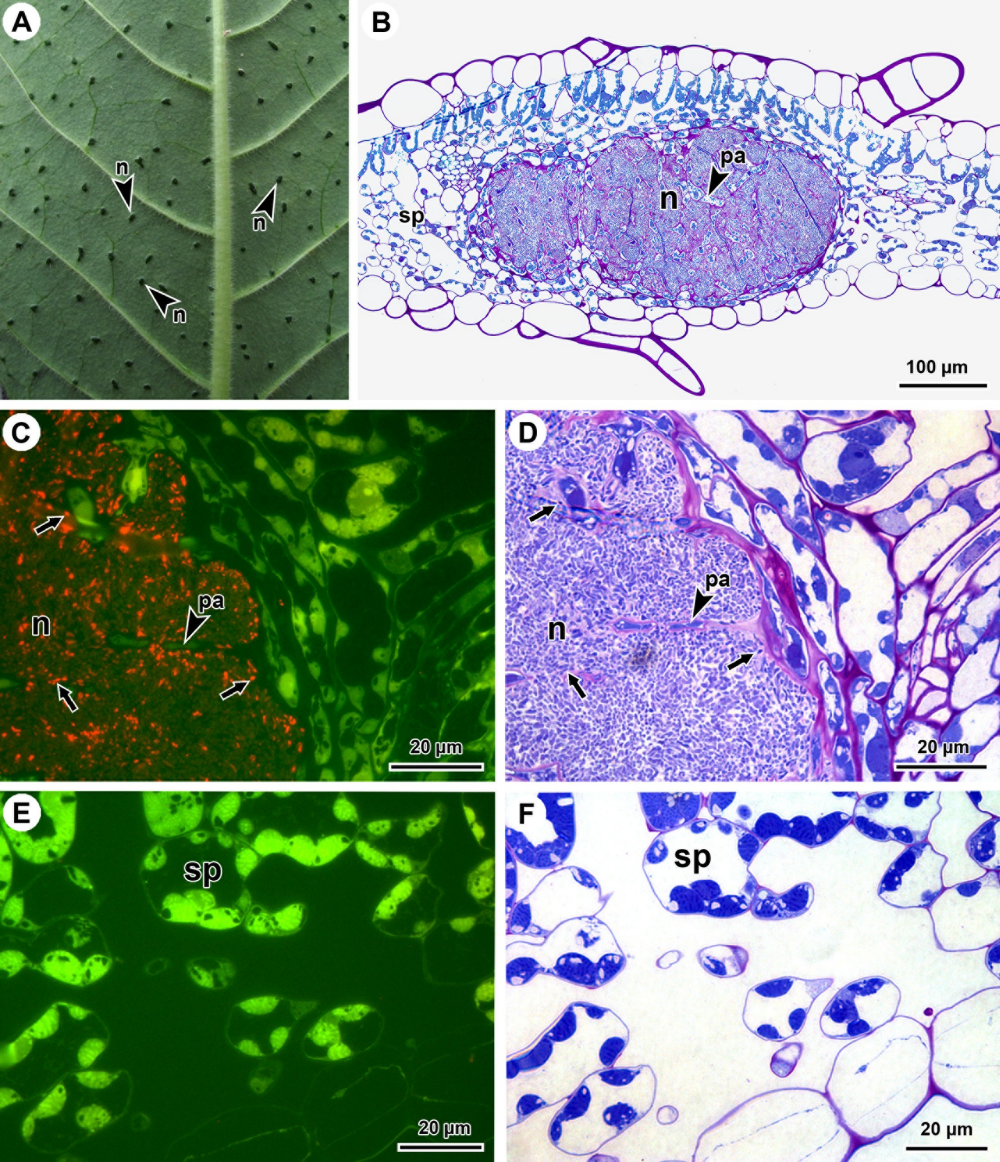
*In situ* detection of *Burkholderia* endophytes in transverse sections of *Psychotria punctata* leaves using FISH. (A) Detail of a leaf showing nodules scattered throughout the abaxial leaf surface. (B) TBO-stained section through a nodule, which contains endophytes, as well as parenchymatous cells. (C) Merged epifluorescence image of the nodule showing FISH-labelled bacteria (red, arrows) and autofluorescence of parenchymatous cells (green). (D) Same section as (C), stained with TBO after FISH labelling, which stains the mucus surrounding the bacteria (arrows correspond with the same bacteria indicated on the fluorescent image (C)). (E) Merged epifluorescence image of the spongy parenchyma showing the intercellular space, in which no endophytes were detected. (F) Same section as (E), stained with TBO after FISH labelling. n, nodule; pa, elongated parenchymatous cells; sp, spongy mesophyll.

The bacterial leaf nodule symbiosis is characterised by macroscopically visible bacterial leaf nodules that are, in case of *P*. *punctata*, randomly distributed in the lamina of the leaves (Fig 1A). The nodules, located in the spongy parenchyma of the mesophyll, are lined by two or three cell layers of compressed mesophyll cells (Fig 1B). FISH labelling allowed identification of the nodule bacteria as *Burkholderia* (Fig 1C). This result indicated that the used protocol and probe allows for *in situ* detection of *Burkholderia* endophytes. The endophytic colony was interspersed with strands of parenchymatous cells (Fig 1D). No endophytes were detected in the extracellular spaces of the spongy parenchyma of the lamina (Fig 1E and 1F).

**Fig 2:**
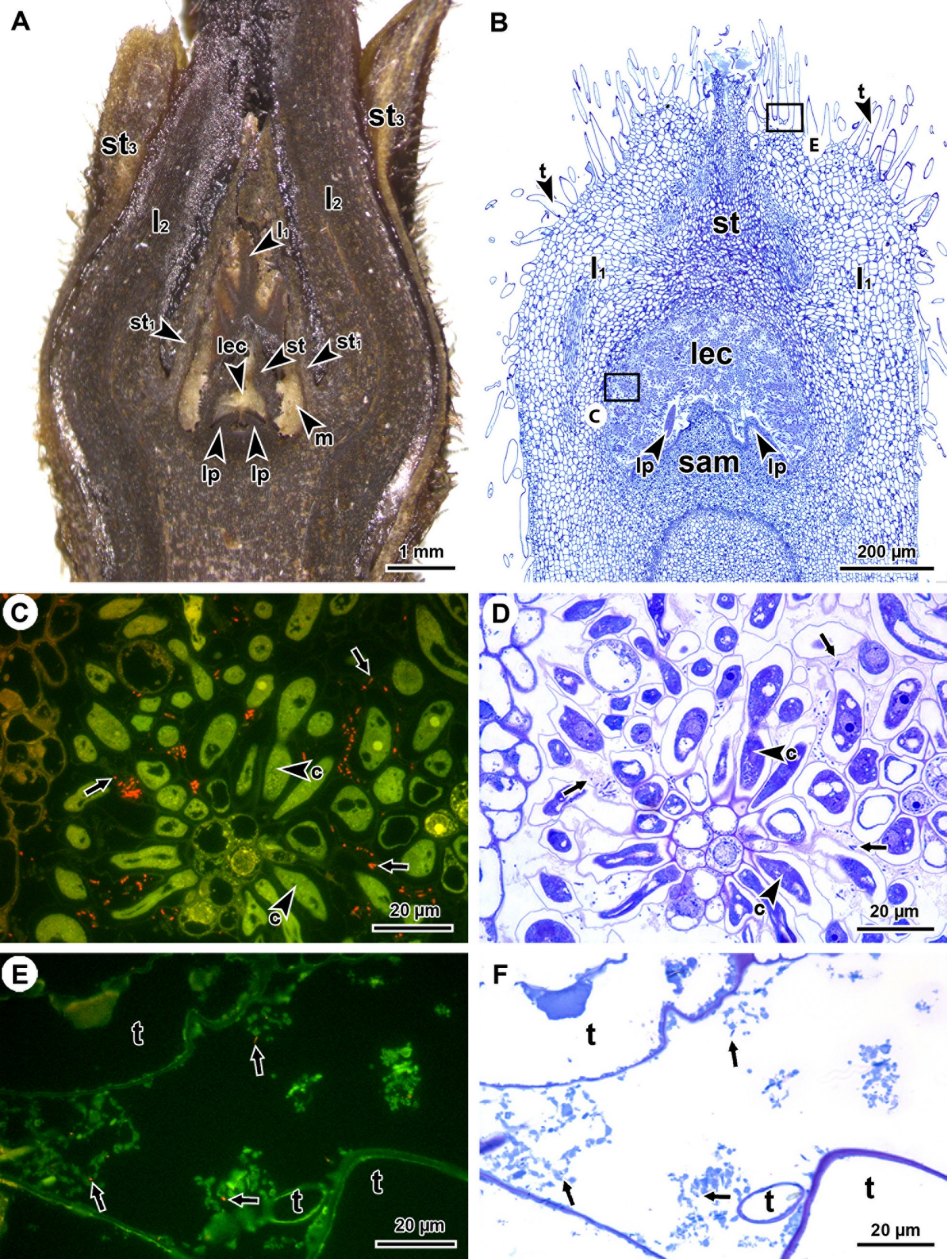
*In situ* detection of *Burkholderia* endophytes in longitudinal sections of *P*. *punctata* vegetative buds using FISH. (A) Overview of an excised vegetative bud revealing the shoot apical meristem and two leaf primordia, and the leaf enclosed chamber (LEC) filled with mucus. The LEC is enclosed by decussate arranged leaf pairs and their interpetiolar stipules. The stipules of the second distalmost node are not visible due to their parallel placement with the sectioning plane. (B) TBO-stained section through the LEC, enclosed by stipules and the leaves of the first distalmost node and the regions visualised in (C and E). (C) Merged epifluorescence image of the LEC (B), showing FISH-labelled bacteria (red, arrows) between the colleters in the mucus. (D) Same section as (C) stained with TBO after FISH labelling. (E) Merged epifluorescence image of the abaxial leaf surface (B), showing *Burkholderia* (red labelling, arrows) in the mucus between the trichomes. (F) Same detail as (E) stained with TBO after FISH labelling. The detected endophytes occur in mucus residue. c, colleters; l1, leaves of first distalmost node; l2, leaves of second distalmost node; lec, leaf enclosed chamber; lp, leaf primordia; m, mucus; sam, shoot apical meristem; st, stipules of the leaf primordia; st1, stipules of the first distalmost node; st3 stipules of third distalmost node; t, trichomes.

In vegetative buds, the leaf enclosed chamber (LEC) is formed by the interpetiolar stipules positioned above the shoot meristem that protect the developing leaf primordia (Fig 2A and 2B). This LEC is further protected by decussately arranged pairs of leaves and their still enclosing interpetiolar stipules (Fig 2A and 2B). In addition to the leaf primordia, multicellular, dendroid trichomes or colleters were detected in the LEC (Fig 2B–2D). These structures were distinguishable from neighbouring cells due to their high level of autofluorescence and their densely stained cytoplasm, which suggests a high cytoplasmic activity, potentially correlated with mucus production (Fig 2C and 2D). At the abaxial side of the enclosing leaves, a different multicellular type of trichomes was observed, characterised by the absence of dense cytoplasmic staining (Fig 2B–2F). *Burkholderia* bacteria were detected in the mucus that surrounds the colleters in the LEC (Fig 2C and 2D). Furthermore, endophytes, yet at a lower abundance compared to the LEC, were also detected in the mucus between the trichomes at the abaxial side of the enclosing leaves (Fig 2E and 2F).

**Fig 3:**
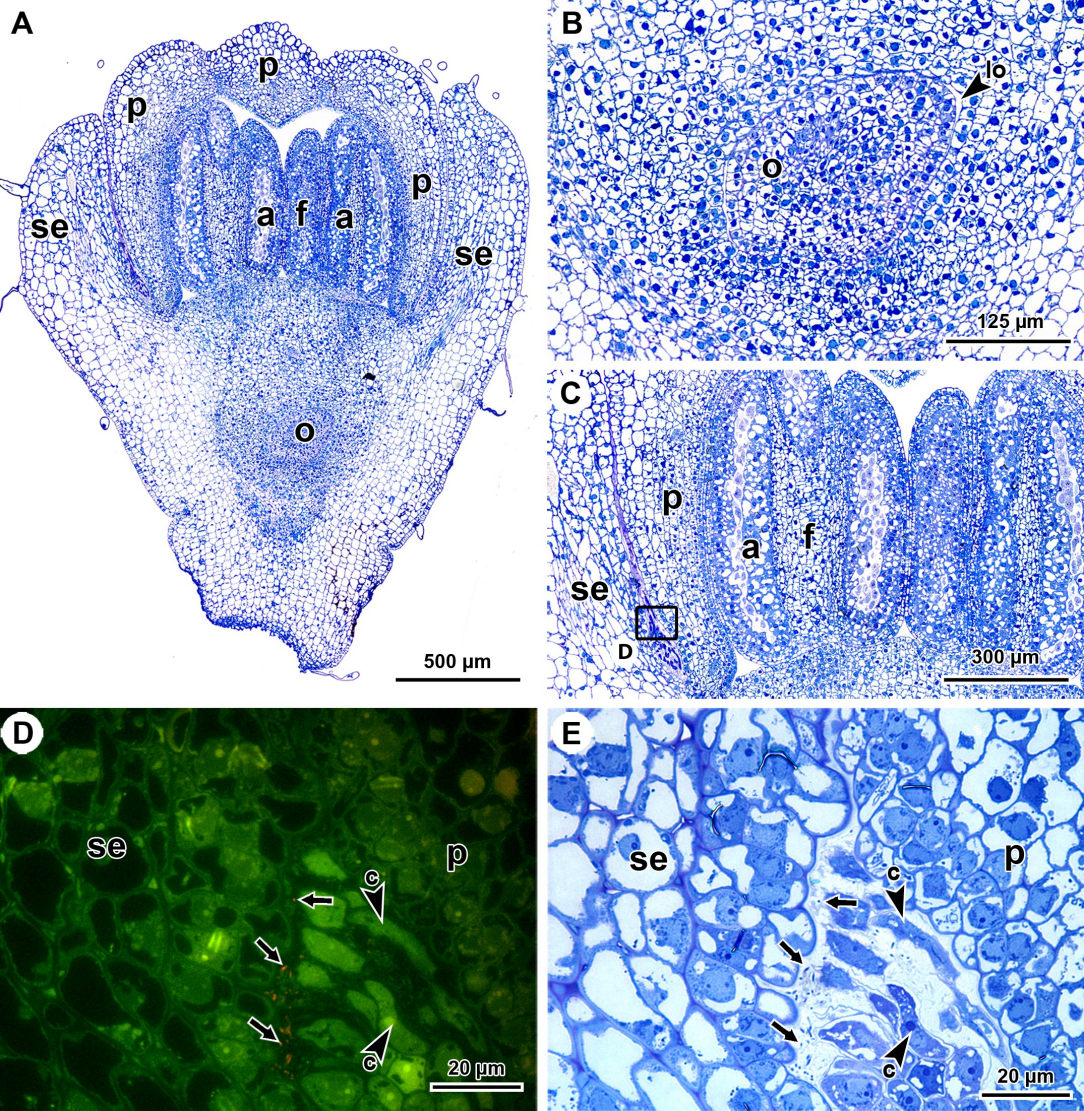
*In situ* detection of *Burkholderia* endophytes in longitudinal sections of *P*. *punctata* flower buds using FISH. (A) TBO-stained section through the flower bud. (B) Detail of (A) showing a locule with ovule. (C) Detail of (A) showing the absence of mucus between the anthers and the location of detail (D) between the sepals and petals. (D) Merged epifluorescence image of the space between the sepals and petals (C), showing FISH-labelled endophytes (red, arrows) between colleters. (E) Same detail as (D) stained with TBO after FISH labelling, visualising mucus between the bacteria and colleters. a, anthers; c, colleters; f, filament; lo, locule; o, ovule; p, petals; se, sepals.

To analyse the presence of the endophytes in flowers, longitudinal sections through developing flower buds were made (Fig 3A). Flowers of *P*. *punctata* are pentamerous, with an inferior gynoecium, bilocular ovaries, uniovulate locules and anatropous ovules (Fig 3A). The observed flower buds were in a late developmental stage, judging by the differentiation state of sepals, petals, and anthers (Fig 3A). Endophytes were not found in the locules (only one of the two locules can be observed) of the gynoecium (data not shown). The developing stamens and style (not shown) are still enclosed by the petals (Fig 3A–3C). FISH labelling only detected endophytes in a mucus-filled space between the sepals and petals (Fig 3D and 3E). At the base of the latter space, colleters with intensely TBO stained cytoplasm were observed (Fig 3C–3E). Although molecular techniques indicated the presence of endophytic DNA in the anthers, no endophytes were detected in these structures by FISH (data not shown).

**Fig 4:**
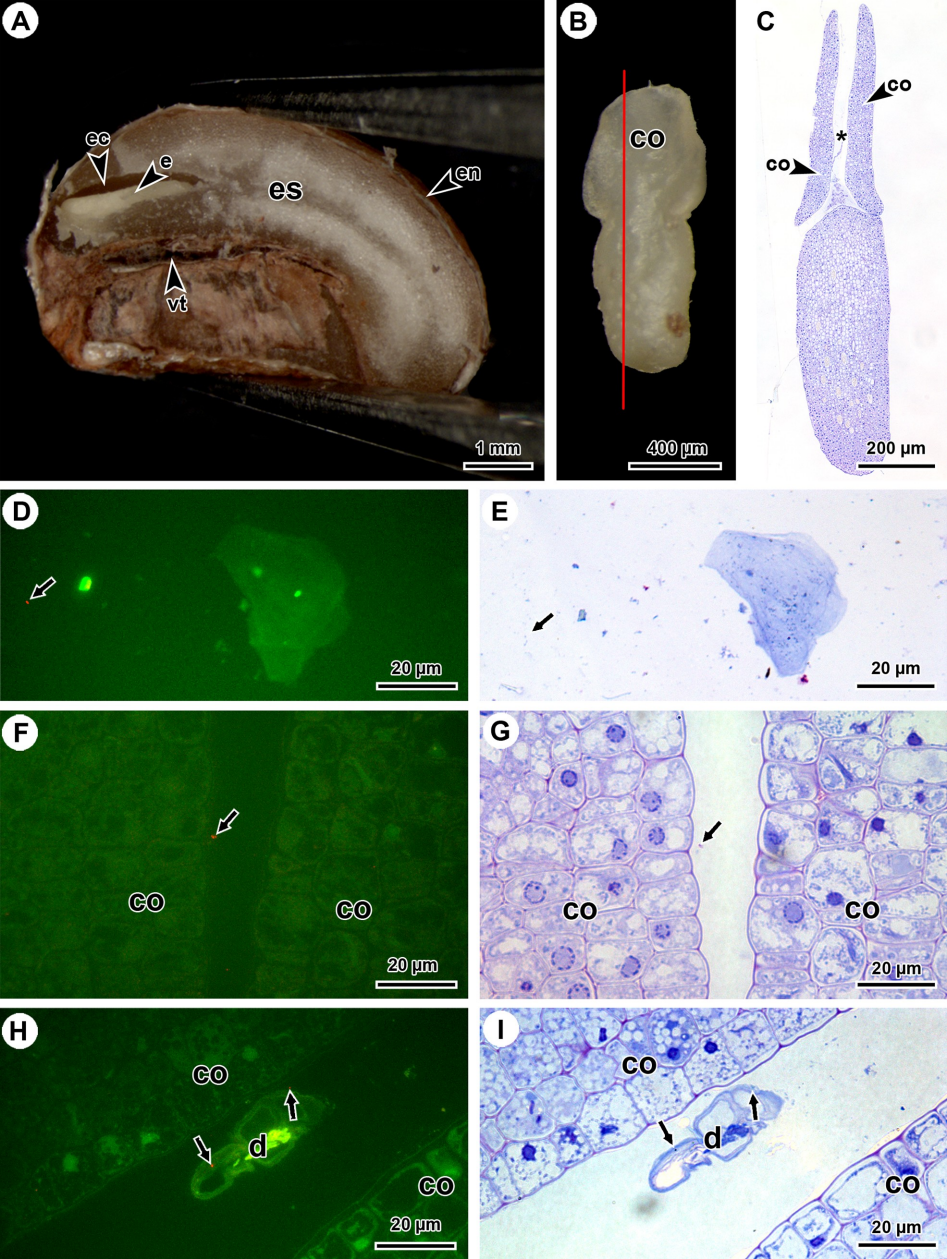
*In situ* detection of *Burkholderia* endophytes in longitudinal sections of *P*. *punctata* embryos using FISH. (A) Overview of a longitudinal section through the pyrene showing the embryo, located in the embryonic cavity, above the ventral intrusion. (B) Overview of an embryo showing the site of the section (red line). (C) TBO-stained section through the embryo of which the sectioning plane is indicated in (B), showing the intercotyledonary space (*). At the shoot meristem, between the cotyledons, mucus is stained between endosperm cell residue. (D) Merged epifluorescence images of the bacterial isolation method, showing a FISH-labelled endophyte (red, arrow). (E) Same detail as (D) stained with TBO after FISH labelling. (F) Merged epifluorescence images of the intercotyledonary space (C; *), showing FISH-labelled endophyte (red, arrow). (G) Same detail as (F) stained with TBO after FISH labelling. (H) Merged epifluorescence images of the intercotyledonary space of a different embryo, showing FISH-labelled endophytes (red, arrows) located between the cotyledons at the outer cell wall surface of degrading endosperm cells. (I) Same detail as (H) stained with TBO after FISH labelling. co, cotyledon; d, degrading endosperm cell; e, embryo; ec, embryonic cavity; en, endocarp; es, endosperm; vt, ventral intrusion.

The fruits of *P*. *punctata* contain one or two hemispherical pyrenes that are characterised by a T-shaped intrusion at the ventral side following the longitudinal axis (Fig 4A). When the pyrene is cut in half, a cavity is observed above the ventral intrusion close to the funiculus in which the embryo is located (Fig 4A and 4B). Mucus was observed in this embryonic cavity and between the cotyledons of the embryo (Fig 4C). Despite the presence of mucus in the embryonic cavity, endophytic DNA was only found in embryos and not in the endosperm or sclerified endocarp samples (Table 1). To assess the presence of endophytes in close association with the embryo, we employed two methods. The first method is based on the collection of bacteria from mucus covering the embryo surfaces (‘bacteria isolation method’). FISH labelling of the extracted residue allowed detection of the *Burkholderia* endophyte (Fig 4D and 4E). In case the endophytes are transmitted vertically, one would expect to detect them in close proximity of the embryonic shoot apical meristem, flanked by the cotyledons. Therefore, longitudinal sections (perpendicular to the surface of the cotyledons) of embryos were investigated using FISH. The second method allowed detection of endophytes–which were also stained with TBO–in the intercotyledonary space of several embryos (Fig 4F–4I and S1E and S1F Fig). In addition to the intercotyledonary space, *Burkholderia* bacteria were also observed at the outer surface of the embryo (S1A–S1D Fig). Endophytes were not detected in the embryonic tissue nor randomly dispersed over the slide.

## Discussion

This study, which combined FISH and PCR techniques, is the first to report and identify the host specific endophyte *Candidatus* Burkholderia kirkii in several locations in the host plant (Table 1). Detection of *Burkholderia* bacteria in the pyrene of *P*. *punctata* demonstrates how these endophytes are vertically transmitted to the next plant generation.

The detection of the endophyte in the leaf enclosing chamber (LEC) above the shoot apical meristem is in line with the findings of previous studies [6,12,19–21]. The location of the latter endophytic colony is an essential part of the symbiotic cycle of the bacterial leaf nodule symbiosis as it enables the transmission of endophytes to the leaves as well as to (axillary) vegetative and reproductive buds. Besides the presence of endophytes, mucus-producing colleters were also present in the LEC. The production of mucus most likely facilitates the passive infiltration of the endophytes towards the leaves during the early leaf developmental stages via stomatal pores [6,19–21]. Endophytes were also detected in mucus residue between the trichomes at the abaxial side of the leaves forming the LEC (Fig 2E and 2F). This can be explained by the formation of successive LECs above the shoot apex. In the LEC, newly developed leaf primordia are completely surrounded by mucus and endophytes. As the latter leaves elongate and cover the LEC, a small amount of mucus and endophytes may still be present at abaxial leaf surfaces. The low abundance of endophytes may be an indirect result of the absence of mucus-producing colleters at the leaf surface and the decreasing mucus production of the colleters at the enclosing stipules, as mucus is necessary to sustain the endophytic colony [20–21,28]. This process might also explain why endophytic DNA was detected in the twig samples, as internodal stem segments also develop in the LEC and endophytic colonies cannot be maintained as mucus-producing trichomes are absent.

The detection of the endophyte in the flower buds, between the sepals and the petals (Fig 3C–3E), supports the findings of earlier studies [6,19]. Moreover, endophytic DNA was confirmed in the gynoecia (Table 1). Although the endophytes were not observed during the microscopic analysis of multiple biological replicates, their presence was suggested by molecular detection. This molecular detection corroborates the observations of von Faber [19] and the current hypothesis [6,12,19–20], which states that the endophytes infiltrate the locules of the gynoecium during early floral development, when the carpels are not yet fused [6,19,20]. This mode of transmission may be possible as Figueiredo et al. [29], in an ontogenetic study of *Psychotria carthagenensis* flowers, observed a (temporary) opening in developing gynoecia with carpels that are not completely fused. There are two hypotheses that explain the infiltration of the ovule by the endophyte: von Faber [19] suggested that the bacteria infiltrate the ovule together with the pollen tube when it fuses with the ovule, while Miller [6], on the other hand, added that, if the embryogenesis of *Psychotria* was apomictic, placental secretion could also facilitate the transmission of the endophyte to the ovule, which in fact has been confirmed for *Ardisia* (Primulaceae). In other mutualistic or parasitic interactions throughout the angiosperms, endophytes may infiltrate the seeds via other pathways including the gametes, the shoot meristem, vascular tissue in the funiculus, the micropyle, or through the seed coat [30–32]. Our results do not unequivocally refute either of these hypotheses, but the absence of endophytes in vascular tissue seems to suggest that the endophytes infiltrate the ovule via the micropyle.

*Candidatus* Burkholderia kirkii was also genetically identified in embryos (Table 1 and S1 Table). FISH labelling supported these results as endophytes were detected in close proximity to the embryo, more specifically in the intercotyledonary space (Fig 4F–4I and S1 Fig). These findings provide strong proof for the presence of endophytes near the embryonic shoot meristem, as suggested by earlier studies [6,19–20]. Although Miller [6] was not able to detect bacterial microorganisms with a transmission electron microscope, we were able to detect *Burkholderia* in the embryonic cavity of five biological replicates (Fig 4 and S1 Fig). The presence of endophytes above the embryonic shoot meristem may be a first and crucial step towards developing successive endophyte-housing LECs as soon as the first leaves appear after germination and vegetative growth is initiated. In other mutualistic or parasitic interactions, seed microbiota is mostly detected near the seed coat and present in grooves, while other bacteria are detected in the endosperm or embryonic tissue [30–34]. In contrast to the endophytes located near the seed coat, the bacteria present in the embryonic tissue are unlikely to be contamination of external microbiota and are vertically transmitted microorganisms [31–34]. In bacterial leaf nodule symbiosis, the close proximity of the endophytes to the embryonic shoot meristem validates the vertical transmission of the endophyte and the obligatory character of the endosymbiosis.

Although endophytic DNA was found in the anthers (Table 1 and S1 Table), complementary FISH experiments on transverse sections through several flower buds did not confirm the presence of endophytes. Transmission of endophytes via pollen (both internally, enclosed within the pollen wall, as externally, attached to the exine) has been reported in other symbiotic interactions, e.g., the bacterial endophyte *Enterobacter cloacae* in Mediterranean pines [35], and endophytic fungi (*Alternaria alternata* and *Cladosporium sphaerospermum*) in forbs [36]. Other studies suggested the possibility of (vertical) endophyte transmission via pollen for bacterial leaf nodule symbiosis as well, but they did not provide conclusive evidence [7,12,37]. Besides transmission via pollen, other modes of transmission, through herbivorous insects or free-living soil bacteria, have been proposed [7,9,12,37]. Our study can neither confirm nor reject these modes of transmission. However, transmission via free-living soil bacteria seems to be less plausible as no endophytic DNA was detected in the roots of the host plant (Table 1).

Even though the number of endophytes in some plant structures appears to be low (Figs 2E and 2F, 4D–4I), these observations are reliable due to the specificity of the FISH-probe and the reproducibility of the observations in multiple biological replicates (Fig 4 and S1 Fig). Comparison of the microscopically observed abundances among the analysed plant structures indicated a decrease from the vegetative apex (Fig 2C and 2D) to the developing flower (Fig 3D and 3E) and the embryo (Fig 4D–4G). This is in line with findings of previous studies investigating the location and abundance of endophytes in *Psychotria* using molecular techniques [8,12] as well as those of other vertically transmitted symbionts, such as *Burkholderia phytofirmans* in *Vitis vinifera* and *Xanthomonas fuscans* in *Phaseolus vulgaris* [38–40]. Based on the above-mentioned decline in endophyte abundance, a low amount might be expected in gynoecia and anthers and this may explain why they were not detected in the latter structures using FISH.

The occurrence of vertical transmission of the endophyte combined with the high specificity and the obligatory nature of the endosymbiosis underline the significance of this interaction for the host species. In other interactions, the endophytes are vertically transmitted due to their importance for seed germination and growth of the seedlings, which increases their survival chance [30–31,41]. Genomic analysis of *Candidatus* Burkholderia kirkii demonstrated the absence of genes or pathways for nitrogen fixation or hormone synthesis that could influence plant growth [8]. However, a specific pathway was detected in the endophytic genome for the production of a C_7_N aminocyclitol, i.e. kirkamide, which protects the host plant against insects [8,37,42–43]. Although our results did not contribute to the functional aspects of the endosymbiosis, the obligate nature of the interaction is reinforced by the confirmation of vertical transmission.

To conclude, our study is the first to unveil the location of the *Burkholderia* endophytes in the seeds, unequivocally confirming vertical transmission. The host specific endophytes were detected in close proximity to the shoot apical meristem of the embryo. This is the first step in the establishment of a key colony in the LEC above the shoot apical meristem, which enables the transmission of endophytes to new leaves and inflorescences. We obtained strong evidence by combining two independent but complementary techniques. The molecular screening enabled the localisation of endophytes in different plant structures, even in not earlier suggested locations. Fluorescent *in situ* hybridisation enabled determination of the *in situ* location of *Burkholderia* endophytes in several plant structures. The results of this study show that adopting a similar approach to other interactions would generate new insights in how endophytes are transmitted.

## Supporting information

S1 Fig *In situ* detection of *Burkholderia* endophytes in longitudinal sections of the three additional *P. punctata* embryos using FISH. (A) Merged epifluorescence images of the outer space of the embryo in close proximity of the cotyledons, showing FISH-labelled endophyte (red, arrow). (B) Same detail as (A), stained with TBO after FISH labelling. (C) Merged epifluorescence images of the outer surface of the embryo close to the cotyledons, showing FISH-labelled endophyte (red, arrow). (D) Same detail as (C), stained with TBO after FISH labelling. (E) Merged epifluorescence images of the intercotyledonary space close to the apical shoot meristem, showing FISH-labelled endophyte (red, arrow). (F) Same detail as (E), stained with TBO after FISH labelling.
(TIF)

S1 Table Detailed information on the identification of the endophytes in the different plant structures of *Psychotria punctata*. List of the detected endophytes, the plant structure from which their DNA was extracted, host plant acquisition numbers of the living collection at the Botanic Garden Meise (BGM), the identity of the endophytes on species level, GenBank accession numbers for 16S rDNA, *gyrB* and *recA*, and the BLAST similarity to the identified species.
(DOCX)
