# Supplementary figures and images for: Detection of Burkholderia in the seeds of Psychotria punctata (Rubiaceae) – Microscopic evidence for vertical transmission in the leaf nodule symbiosis
Source: PLoS One. 2018 Dec 14;13(12):e0209091. doi: 10.1371/journal.pone.0209091 (PMC6294375; doi:10.1371/journal.pone.0209091)

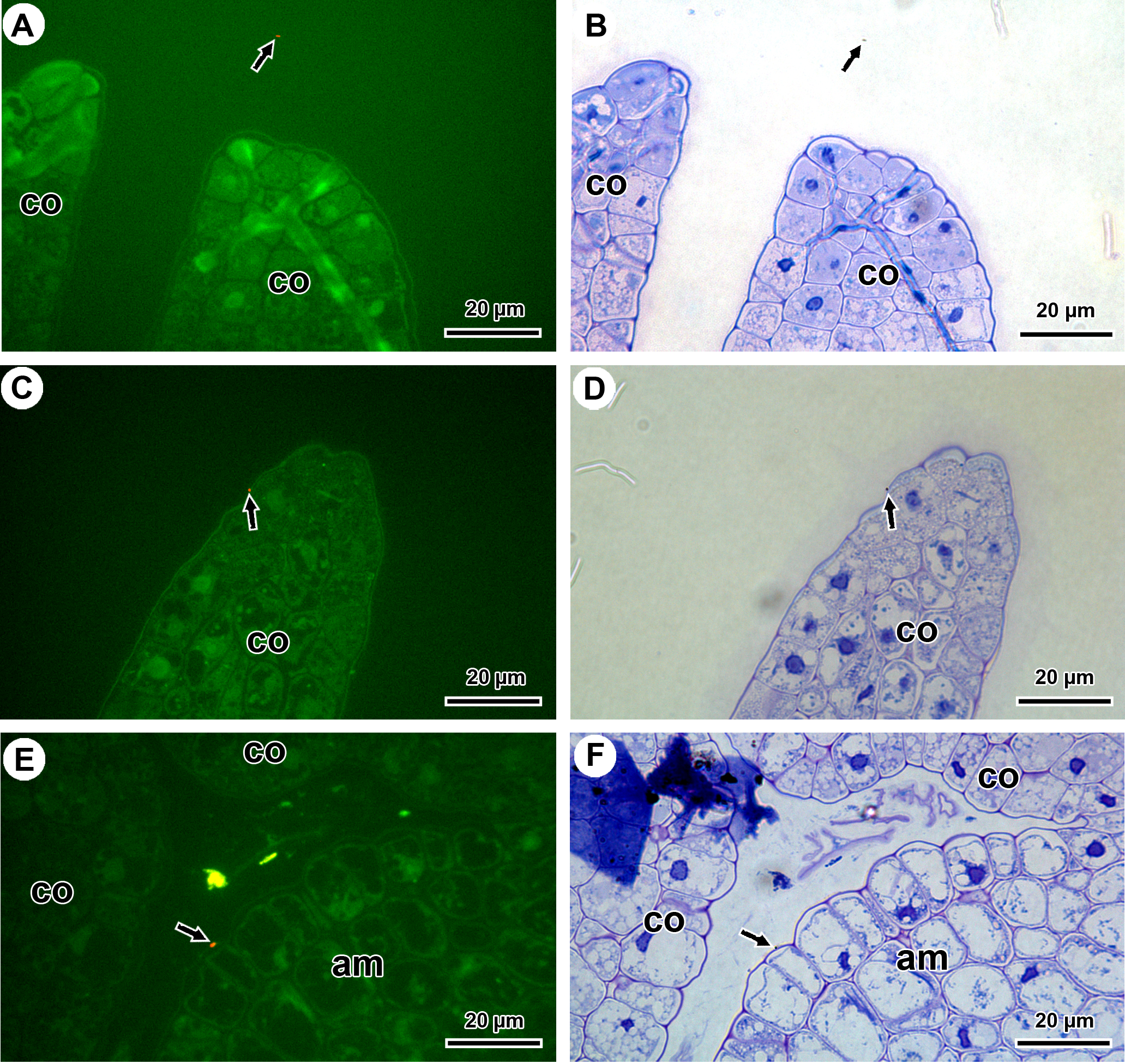

Supplement: S1 Fig — (A) Merged epifluorescence images of the outer space of the embryo in close proximity of the cotyledons, showing FISH-labelled endophyte (red, arrow). (B) Same detail as (A), stained with TBO after FISH labelling. (C) Merged epifluorescence images of the outer surface of the embryo close to the cotyledons, showing FISH-labelled endophyte (red, arrow). (D) Same detail as (C), stained with TBO after FISH labelling. (E) Merged epifluorescence images of the intercotyledonary space close to the apical shoot meristem, showing FISH-labelled endophyte (red, arrow). (F) Same detail as (E), stained with TBO after FISH labelling. (TIF) [file pone.0209091.s001.tif]
