# Supplementary material for: Detection of Burkholderia in the seeds of Psychotria punctata (Rubiaceae) – Microscopic evidence for vertical transmission in the leaf nodule symbiosis
Source: PLoS One. 2018 Dec 14;13(12):e0209091. doi: 10.1371/journal.pone.0209091 (PMC6294375; doi:10.1371/journal.pone.0209091)
Supplement: S1 Table — List of the detected endophytes, the plant structure from which their DNA was extracted, host plant acquisition numbers of the living collection at the Botanic Garden Meise (BGM), the identity of the endophytes on species level, GenBank accession numbers for 16S rDNA, gyrB and recA, and the BLAST similarity to the identified species. (DOCX) [file pone.0209091.s002.docx]

**S1 Table. Detailed information on the identification of the endophytes in the different plant structures of *Psychotria punctata******.*** List of the detected endophytes, the plant structure from which their DNA was extracted, host plant acquisition numbers of the living collection at the Botanic Garden Meise (BGM), the identity of the endophytes on species level, GenBank accession numbers for 16S rDNA, *gyrB* and *recA*, and the BLAST similarity to the identified species.

|  | **Plant structure** | **Acquisition number living collection BGM** | **Endophyte identity** | **GenBank accession number** | | | | | |
| --- | --- | --- | --- | --- | --- | --- | --- | --- | --- |
|  |  |  |  | 16S rDNA | BLAST similarity | *gyrB* | BLAST similarity | *recA* | BLAST similarity |
| 1 | Leaves | 19536779 | *Candidatus* B. kirkii | MK132783 | 100 % | MK132797 | 100 % | – | – |
| 2 | Leaves | 20010513-92 | *Candidatus* B. kirkii | MK132782 | 100 % | MK132798 | 100 % | MK132825 | 100 % |
| 3 | Leaves | 20021526-47 | *Candidatus* B. kirkii | MK132780 | 100 % | MK132804 | 100 % | MK132824 | 100 % |
| 4 | Vegetative buds | 19536779 | *Candidatus* B. kirkii | MK132789 | 100 % | MK132796 | 100 % | MK132821 | 100 % |
| 5 | Vegetative buds | 20010513-92 | *Candidatus* B. kirkii | MK132790 | 100 % | MK132815 | 100 % | MK132820 | 100 % |
| 6 | Vegetative buds | 20021526-47 | *Candidatus* B. kirkii | MK132792 | 99.8 % | MK132794 | 100 % | MK132819 | 100 % |
| 7 | Flower buds | 20001943-58 | *Candidatus* B. kirkii | MK132785 | 100 % | MK132802 | 100 % | MK132828 | 100 % |
| 8 | Flower buds | 19536779 | *Candidatus* B. kirkii | MK132788 | 100 % | MK132806 | 100 % | MK132827 | 100 % |
| 9 | Flower buds | 1995273-22 | *Candidatus* B. kirkii | MK132791 | 100 % | MK132803 | 100 % | MK132826 | 100 % |
| 10 | Anthers | 20001943-58 | *Candidatus* B. kirkii | MK132773 | 99 % | MK132816 | 99.5 % | MK132836 | 100 % |
| 11 | Anthers | 19536779 | *Candidatus* B. kirkii | MK132774 | 100 % | MK132800 | 99.7 % | MK132834 | 100 % |
| 12 | Anthers | 1995273-22 | *Candidatus* B. kirkii | – | – | MK132811 | 100 % | MK132835 | 100 % |
| 13 | Gynoecia | 20001943-58 | *Candidatus* B. kirkii | MK132786 | 100 % | MK132807 | 100 % | MK132831 | 100 % |
| 14 | Gynoecia | 19536779 | *Candidatus* B. kirkii | MK132793 | 99.4 % | MK132814 | 100 % | MK132829 | 100 % |
| 15 | Gynoecia | 1995273-22 | *Candidatus* B. kirkii | MK132787 | 100 % | MK132808 | 100 % | MK132830 | 100 % |
| 16 | Embryos | 19536779 | *Candidatus* B. kirkii | MK132775 | 100 % | MK132809 | 100 % | MK132837 | 99.8 % |
| 17 | Embryos | 19951273-22 | *Candidatus* B. kirkii | MK132776 | 100 % | MK132813 | 100 % | MK132833 | 100 % |
| 18 | Embryos | 20001943-58 | *Candidatus* B. kirkii | MK132777 | 100 % | MK132795 | 99.5 % | MK132832 | 100 % |
| 19 | Twigs (second internode) | 19536779 | *Candidatus* B. kirkii | MK132778 | 100 % | MK132799 | 100 % | MK132817 | 99.8 % |
| 20 | Twigs (second internode) | 20010513-92 | *Candidatus* B. kirkii | **MK132781** | 100 % | – | – | – | – |
| 21 | Twigs (second internode) | 20021526-47 | *Candidatus* B. kirkii | – | – | MK132810 | 100 % | MK132818 | 100 % |
| 22 | Twigs (lignifying) | 19536779 | *Candidatus* B. kirkii | MK132784 | 100 % | MK132805 | 100 % | MK132823 | 100 % |
| 23 | Twigs (lignifying) | 20010513-92 | *Candidatus* B. kirkii | – | – | MK132812 | 99.8 % | – | – |
| 24 | Twigs (lignifying) | 20021526-47 | *Candidatus* B. kirkii | MK132779 | 100 % | – | – | MK132822 | 100 % |
